# How to promote universities’ research and development into green agricultural products – A tripartite evolutionary game analysis

**DOI:** 10.1371/journal.pone.0342240

**Published:** 2026-02-10

**Authors:** Lin Xiong, Luyao Chang, Yiyi Luo, Hansheng Wu, Yuchao Wang, Huile Jin

**Affiliations:** 1 Institute of Higher Education, Wenzhou University, Wenzhou, China; 2 Business School, Wenzhou University, Wenzhou, China; 3 College of Biology, Hunan University, Changsha, China; 4 School of Economics and Management, Yunnan Minzu University, Kunming, China; 5 Key Lab of Advanced Energy Storage and Conversion, College of Chemistry and Materials Engineering, Wenzhou University, Wenzhou, China; USTC: University of Science and Technology of China, CHINA

## Abstract

Research into green agricultural products significantly enhances the sustainable development of China’s agriculture. These products ensure food safety, meet quality demands, reduce pollution, and advance agricultural green transformation. Utilising evolutionary game theory, this study analyses the interactions among the government, universities, and consumers in the research and development (R&D) on green agricultural products. Results show that under specific conditions, strategic interactions evolve toward stable states: (1,1,0) or (1,1,1). Secondly, critical factors affecting universities’ R&D demonstrate threshold effects. Third, government incentive measures have a significant impact on the R&D willingness of universities and the choices of consumers drives the enthusiasm of universities in making R&D decisions. Policy effectiveness and the outcomes of universities’ R&D subsequently constrain consumer choices. Short-term government incentives are crucial for promoting universities’ green agricultural R&D. Long-term sustainability, however, requires integrating market-driven incentives with government policies to sustain universities’ R&D strategies. This work offers theoretical and practical guidance for governmental policy-making, universities’ R&D strategies, and the upgrading of consumer behaviour. These findings critically support agriculture’s green and sustainable development.

## 1. Introduction

Green agriculture incorporates the principles of sustainable development into all stages of agricultural production. It is founded on the tenets of ecological sustainability, environmental protection, and efficient resource use. This approach seeks to preserve ecosystem stability and harmonise agricultural economic, ecological, and social benefits by refining production techniques and minimising polluting emissions. Its central objective is to propel the industry’s transition towards sustainability and to establish an agricultural system that is green, low-carbon, circular, and efficient [1]. In China, the advancement of green agriculture has emerged as a pivotal strategy for modernising the agricultural sector, safeguarding food security, and fostering sustainable development. In recent years, the Chinese government and associated ministries have released numerous guidelines and implementation criteria to vigorously propel a coordinated shift towards ecological farming practices. A key document, the “Guiding Opinions on Accelerating the Comprehensive Green Transformation of Agriculture and Promoting Rural Ecological Revitalisation”, was published by the Ministry of Agriculture and Rural Affairs. This policy outlines objectives to establish a preliminary green, low-carbon, circular agricultural industrial system by 2030. Furthermore, it aims to largely establish a green agricultural production model by 2035. These measures are intended to advance rural ecological revitalisation and foster a harmonious coexistence between humanity and nature. The broad adoption of these green principles has significantly elevated agricultural production standards, optimised resource allocation, and enhanced management and service efficiency. Consequently, this has fortified the sector’s competitiveness and sustainability, vigorously advancing the transition of Chinese agriculture to an ecologically sound paradigm.

With the acceleration of green agricultural infrastructure construction, research and development on green agricultural products in universities is still in its infancy. In the R&D stage of green agricultural products, although some universities have a solid scientific research foundation, there are still significant shortcomings in practice: first, there is a lack of accumulated practical experience, leading to a disconnect between theory and application; second, there is insufficient autonomous driving force for the innovation of germplasm resources and the R&D of green and efficient agricultural inputs, resulting in low technology conversion efficiency; third, the response mechanism to diverse market demands is lagging, making it difficult to quickly launch adapted products [2]. China’s “Rural Revitalization Strategy” clearly states the need to “support universities in leveraging their research and talent advantages, deepen the integration of industry, academia, and research, and serve the green development of agriculture”, which fully reflects the country’s emphasis on the core role of universities in promoting sustainable agricultural development and facilitating the R&D of green agricultural products. However, constrained by various issues such as the agricultural development foundation in different regions, differences in industrial demands, and the uneven distribution of research resources within universities, many regions still lack systematic research support and talent empowerment from universities in the breakthrough of key technologies for green agricultural products and the promotion of sustainable agricultural innovation models. Therefore, encouraging the active involvement of universities in sustainable production represents a crucial approach to transitioning towards ecological agriculture. Furthermore, universities’ engagement significantly contributes to Chinese agriculture’s green and sustainable development [3]. Universities integrate agricultural researchers and students who collaborate directly with farmers in production settings, enabling the development of context-specific sustainable technologies that address local agricultural requirements. Through regular engagement, they promote ecological awareness and guide appropriate agricultural development perspectives among farming communities. It can be said that, through the joint efforts of universities and Science and Technology Backyards, an important platform for sustainable agriculture has been established, one that deeply integrates technological innovation with practical application [4].

In the field of green agricultural product R&D, how to establish an innovative system that deeply integrates technological theory and practice has become a core issue in current research on sustainable agricultural development [5].Compared with other agricultural organisational forms, universities have demonstrated distinctive comparative advantages and notable innovation outcomes during the development phase of green agricultural product R&D. From the perspective of technological innovation, universities take ecological protection [6] and cost control [7] as dual objectives, actively exploring sustainable development pathways for agriculture. Taking the Yuhang Honey Pear Science and Technology Backyard as an example, this entity has established an innovation consortium led by enterprises and coordinated between universities and local governments, comprehensively applying soil-tested fertilisation, horticultural technology upgrades, and a green pest control technology system to achieve the green transformation of the entire honey pear cultivation process, successfully cultivating high-quality agricultural products. According to statistics, this model has expanded the planting area in the region to more than 8,000 mu, with an annual output value exceeding 240 million yuan, becoming a typical model for green agricultural development in Zhejiang Province. As a pilot platform for agricultural technological innovation, the Science and Technology Backyard, through a mechanism of on-site service by scientific researchers, has adapted cutting-edge achievements such as intelligent greenhouse management systems and biological control technologies to local conditions and promoted them to surrounding areas [8].This integrated “industry-academia-research-application” model not only cultivates professionals with both theoretical knowledge and practical skills for the agricultural sector but also injects sustained momentum into the development of green agriculture [9]. Nevertheless, theoretical disagreements persist within academia concerning universities’ role in researching, developing, and producing sustainable agricultural products. Certain academics emphasise the government’s dominant macro-regulatory function in agricultural sustainability, maintaining that policy guidance and resource allocation primarily drive technological diffusion [10]; an alternative perspective highlights market incentive mechanisms affecting consumer demand [11], contending that developing consumer-oriented sustainable products can enhance universities’ innovation motivation. This theoretical divergence reflects the complex nature of universities’ function in sustainable agricultural development and underscores the necessity for thorough examination of their part in developing and producing ecological agricultural products.

Existing research provides valuable insights into the sustainable development of green agriculture, but studies on the R&D of green agricultural products by universities remain insufficient. Current research primarily focuses on the agricultural production processes of smallholder farmers, emphasising the impacts of traditional agriculture on environmental, social, and governance dimensions [12]. University-based Science and Technology Backyards integrate universities’ scientific research resources with agricultural production practices to develop and promote green planting technologies, reduce the use of pesticides and chemical fertilisers, thereby improving soil quality, reducing pollution from agricultural production, and further enhancing the quality of agricultural products, which contributes to the sustainable development of agricultural ecosystems and increases farmers’ income. They have established a collaborative platform between universities’ scientific research and front-line production practices and conduct problem-oriented research based on regional agricultural ecological characteristics. On the one hand, universities utilise material and energy theory to convert waste materials such as straw and manure into organic fertilisers through biotechnology, forming a closed-loop system of “production-processing-return to field”.This reduces external dependence and controls costs while ensuring nutrient supply for green production [13]. On the other hand, universities focus on controlling non-point source pollution [14], optimising fertiliser and pesticide application techniques to reduce pollution at the source, and promoting changes in farmers’ production methods through training and outreach to foster the development of green industrial clusters [15], thereby exploring new pathways for sustainable agricultural development.However, the academic community has yet to establish a systematic framework for understanding the mechanisms, practical pathways, and contributions of universities to the R&D of green agricultural products, as well as their impact on agricultural green sustainable development. This limitation has, to some extent, constrained the theoretical innovation and practical application of this model.

This study is grounded in the development trends of green agriculture and delves into the core role and operational mechanisms of universities in the R&D of green agricultural products, offering significant theoretical contributions and practical value. At the theoretical level, through the construction of a systematic analytical model, this study provides a detailed breakdown of how universities drive technological innovation breakthroughs, integrate various resource elements, and ultimately enhance ecological benefits in the process of promoting the transformation of green agriculture. In terms of technological integration and innovation, this study explains the driving forces behind the R&D of green agricultural products. It further reveals the interactive logic and dynamic balance among multiple stakeholders---including the government, universities, and consumers---thereby opening up new directions for research on sustainable green agriculture. In addition, by clarifying the positioning and functions of universities in the entire agricultural industry chain, this study improves the theoretical framework for agricultural science and technology innovation and promotion, providing new research ideas for the promotion of green agriculture by universities. In terms of practical application, the findings of this study can provide a scientific basis for the government to formulate policies that incentivise green agricultural development, promote the rational allocation of resources, and drive the coordinated development of universities and green agriculture. They can also enhance society’s understanding of green agriculture, increase consumer trust in green agricultural products, regulate market development, and reduce the pressure of agricultural production on the ecological environment through the demonstration effect of universities, thereby achieving the organic unity of agricultural economic, ecological, and social benefits and providing a strong guarantee for rural ecological revitalisation and the construction of an ecological civilisation.

The structure of this paper is organised as follows: Section 2 establishes the theoretical foundation and identifies the key evolutionary agents. Subsequently, Section 3 delineates the model’s assumptions, conducts the analysis, and summarises the findings. It also investigates potential equilibrium states alongside their respective stability conditions. Following this, Section 4 details the simulation experiments and provides a comparative analysis of critical sensitivity variables. Section 5 then examines the implications of these findings, situating them within a broader context and discussing their practical applications. Finally, Section 6 concludes the paper and proposes recommendations derived from the study’s outcomes.

## 2. Materials and methods

### 2.1. Application of evolutionary game theory

Evolutionary game theory provides a fundamental analytical framework for examining strategic decisions among interacting agents within dynamic systems. This approach relaxes conventional perfect rationality assumptions, offering robust methodologies for analysing bounded rationality and longitudinal behavioural evolution. The framework excels in modelling complex interactions, tracking collective behavioural evolution, and revealing mechanisms underlying stable strategy formation [16]. When analysing the behavioural decision-making of agents with bounded rationality, evolutionary game models demonstrate significant theoretical advantages, as they essentially capture the dynamic process by which agents achieve equilibrium through trial and error and strategy adjustments. In exploring the interactive relationship among the government, universities, and consumers in promoting the R&D of green agricultural products under conditions of bounded rationality, this study will comprehensively apply evolutionary game theory and simulation techniques for an in-depth analysis.

The core theoretical framework of evolutionary game theory comprises three key elements: payoff matrices, evolutionary stable strategies, and replicator dynamics equations. Payoff matrices are used to quantify the distribution of payoffs among different game agents under various strategy combinations, describing the payoffs obtained by different participants when selecting different strategies [17]. Evolutionarily stable strategies refer to stable strategy forms that dominate a population and can resist invasion by other strategies [18]. Replicator dynamics equations construct dynamic system models to analyse the evolutionary trajectories of population strategies and the formation process of stable strategies [19].

The green transformation of agriculture constitutes an intricate system defined by interactions and adaptations among multiple participants, presenting substantial and specialised difficulties. Evolutionary game theory serves as a particularly valuable methodological approach for examining these challenges. This theory offers a structured, scientific framework for comprehending participant interactions within specific settings. It further elucidates how these agents adjust their strategies based on behavioural outcomes. Within this framework, the strategic decision of universities on whether to actively engage in the R&D of green agricultural products can be modelled as two discrete choices, each linked to a separate evolutionary path. By analysing the interaction between these strategies using replicator dynamics or simulation methods, we can identify which strategy proves more advantageous in adapting to the environmental conditions, thus predicting its dominance in the evolutionary process. Furthermore, evolutionary game theory is particularly well-suited for elucidating the emergent cooperative and competitive behaviours that arise among participants. Therefore, this theoretical framework provides an indispensable analytical tool for promoting green sustainable agricultural development, helping participants understand their interactions and adaptation issues from a professional perspective, and subsequently formulate optimal strategies based on the analysis results, thereby providing a robust theoretical foundation for sustainable agricultural development practices.

### 2.2. The main participants in this game

By taking root in rural areas and serving farmers, universities empower green agriculture with technology, becoming an important engine for promoting China’s green agricultural transformation and high-quality development [20]. Therefore, the R&D of green agricultural products by universities has become a critical issue. Achieving this requires the joint participation of the government, universities, and consumers.

Government: Within the overarching framework for green agriculture, which carries the dual objectives of ecological conservation and sustainable farming, the government holds an indispensable position. To advance this sector, governing bodies face a critical choice regarding the implementation of incentive policies. Current Chinese policies designed to encourage universities to lead sustainable agricultural development and elevate consumer consumption patterns primarily focus on two measures. First, transfer payments are chiefly directed towards research initiatives and R&D subsidies. This subsidy mechanism, often price-based, mitigates the financial pressures faced by universities involved in green agricultural R&D. Concurrently, it mitigates opportunistic behaviours and moral hazards, including subsidy fraud [21]. This encourages universities to engage in technological research and application, striving to become a frontier for green agricultural technological innovation and practice. On the other hand, publicity and education campaigns aim to foster a shift in consumer preferences, guiding the market towards adopting new consumption patterns. This, in turn, stimulates consumption-led investment and innovation, thereby providing sustained momentum for the sustainable development of green agriculture from the demand side [22]. The two complement each other, jointly providing strong impetus for the sustainable development of green agriculture and driving the agricultural industry towards a greener, more efficient, and sustainable direction. However, the extent of government R&D subsidies and publicity is difficult to gauge, and strong policy incentives also mean increased fiscal expenditures, placing additional pressure on government finances. Therefore, the government must interact with Science and Technology Backyards and consumers to find the optimal decision.

Universities: At a critical stage in the accelerated advancement of agricultural modernisation, universities have emerged as an innovative entity for agricultural research. Through their specialized operational systems, universities are playing a key role in driving the progress of green agriculture [3]. When deciding whether to leverage universities to promote the sustainable development of green agriculture, the costs and benefits of universities’ R&D of green agricultural products are critical factors that must be considered. After paying related costs, universities can obtain government policy subsidies and investments from enterprises and institutions through their operational mechanisms. However, the high R&D costs make it uncertain whether the generated benefits can cover the costs. Consequently, owing to the profit-maximising imperative inherent to economic organisations, leaders consequently seek increased subsidies and elevated agricultural product prices to mitigate unforeseen operational expenditures [23].

Consumers: With the development of the economy and society, consumer demand has shifted from “eating enough” to “eating well”, and consumers’ preference for green agricultural products is the long-term support for the sustainable development of green agriculture [24]. Through technological innovation and promotion, universities can help farmers increase the yield and quality of agricultural products, providing consumers with higher-quality, safer, and more diverse products to meet their demand for a high-quality life. Therefore, consumers face the choice of whether to upgrade their consumption. If consumers have a high willingness to consume and a high level of recognition for the products or technologies promoted by universities, market demand will be high, which will encourage universities to expand their scale, strengthen R&D, it may lead to universities adjusting their strategies or facing development challenges.

**Fig 1:**
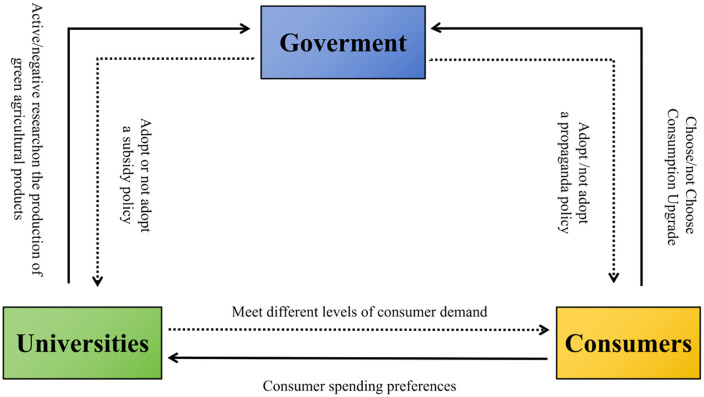
Logical relationships in the tripartite evolutionary game model.

Governments, universities, and consumers base their decisions upon cost-benefit analyses. Although their interests may converge in specific domains, they choose to collaborate based on distinct underlying rationales. This indicates the presence of ongoing strategic interdependence between these actors. These strategic relationships are depicted in Fig 1.

## 3. Calculation

### 3.1. Model assumptions

Considering the present context of universities’ engagement with the R&D of green agricultural products, the following assumptions are formulated concerning the behavioural patterns and benefits of the three parties within this evolutionary model:

Assumption 1: The government, universities, and consumers operate under conditions of incomplete information and are all limitedly rational, each seeking to maximise their own interests. The Tripartite engages in an evolutionary game.

Assumption 2: The government, universities, and consumers face a set of strategic decisions. The government must decide whether to adopt or reject incentive policies. Adoption entails providing subsidies to universities for green agricultural R&D, while also promoting consumption upgrades among consumers. The probability of the government adopting these policies is denoted as x; consequently, the probability of rejecting them (deeming further incentives unnecessary) is 1-x. For universities, the probability of engaging in the R&D of green agricultural products is y, making the probability of rejection 1-y. Similarly, the probability that consumers upgrade their consumption is z, whereas the probability of them opting for traditional consumption is 1-z.

Assumption 3: Upon implementing incentive policies, the government must encourage universities to research and produce green agricultural products, while also promoting consumption upgrades among consumers. Specifically, the government allocates subsidies to universities for green agricultural R&D. Concurrently, it educates consumers and promotes consumption upgrades through public awareness campaigns. The total cost of subsidies for universities is α*Cg₁, and the total cost of consumer publicity subsidies is β*Cg₂, where α is the subsidy coefficient for R&D equipment, and *β* is the publicity coefficient for consumer advertising campaigns. Increased willingness of universities to produce green agricultural products and the widespread adoption of digital agriculture in China---the government will realise the benefit Rg, representing sustainable agricultural development. Conversely, if the government declines to provide policy incentives, its inaction will result in increased management costs, denoted as *Cg₃* (*Cg₃ > *α*Cg₁ + β*Cg₂) [25].

Assumption 4: Universities derive benefits from engaging in the R&D of green agricultural products. These benefits include direct R&D subsidies from the government, quantified as α*Cg₁, as well as indirect gains from improved product quality and consumption upgrades. If a university actively engages in the R&D of green agricultural products, its resultant income is *Rm₁*. By contrast, The benefits of passive involvement in green agricultural product R&D is Rm₂. Here, Rm₁ = i*P₁*Q₁, where i represents consumer preference for high-quality goods, *P₁* denotes the price of green agricultural products, and *Q₁* is their output. If a university is reluctant to engage in R&D, its income is Rm₂, given by Rm₂ = P₂*Q₂ (*Rm₂ < Rm₁*). Here, *P₂* and Q₂ represent the price and quantity of the agricultural product, respectively, under the scenario of passive R&D by universities.(This type of agricultural product is uniformly referred to as ordinary agricultural products.) Universities must also account for the costs of green R&D, such as those associated with technology acquisition, equipment maintenance, and depreciation. Both pre-R&D production costs (*Cf₁*) and post-R&D production costs (*Cf₂*) must be considered, with *Cf₁ > Cf₂.* In China, the application process for R&D subsidies is cyclical. Furthermore, markets are often led by technological early adopters, which can constrain profit-maximising opportunities for universities engaged in green production. Surveys show that universities reluctant to engage in R&D have often incurred losses in the absence of government subsidies. This study conceptualizes these losses as a regret cost (*G*), formally defined as *G = *α*Cg₁ [26].

Assumption 5: Rooted in Maslow’s hierarchy of needs theory, individuals seek social recognition for their behaviour and achievements to fulfil their esteem needs. Government-led publicity campaigns shape social norms, which in turn foster a sense of social identity among consumers who choose to upgrade their consumption [27]. Consequently, intensified government publicity can shift consumer attitudes, leading those who opt for consumption upgrades to incur the cost *Rm₁* of purchasing green agricultural products. However, if universities refuse to engage in the R&D of green agricultural products, a supply shortage arises. Consequently, consumers seeking to upgrade their consumption face a market lacking high-quality products. Given that agricultural products are largely necessities, these consumers will ultimately resort to traditional consumption, incurring the lower cost *Rm₂* of ordinary agricultural products. Alternatively, some consumers with strong preferences for high-quality goods may independently seek out green agricultural products despite the shortage. This study defines the associated expenses as search costs (*Sc*). Furthermore, the social norms cultivated by government publicity provide consumers who upgrade their consumption with a sense of social recognition. This psychological satisfaction and perceived social value are denoted as *S* (*S* > *Rm₁-Rm₂*).

**Fig 2:**
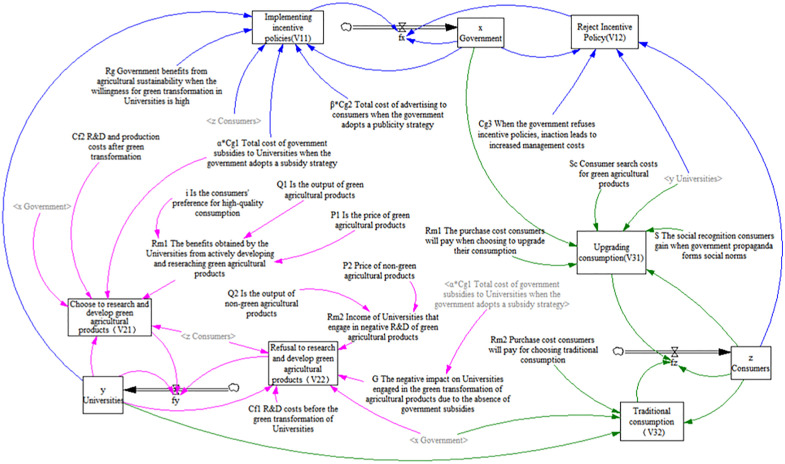
This figure provides formal definitions for all key variables used in the model and delineates the conceptual relationships between them.

Building upon the preceding analysis, Table 1 summarizes the key assumptions and definitions of all variables incorporated in the evolutionary game model. Furthermore, the interrelationships and conceptual framework linking these variables are depicted in Fig 2 (blue lines indicate the relationship between the variable and government decisions, pink lines indicate the relationship between the variable and the universities’ decisions, and green lines indicate the relationship between the variable and consumer decisions). The payoff matrices for the government, universities, and consumers are presented in Table 2.

**Table 1:** Assumptions and meanings of relevant variables in the evolutionary game model.

Variable	Meaning
*Rm₁*	The benefits obtained by the universities from activelyresearching and developing green agricultural products, Rm₁ = i*P₁*Q₁.
*i*	Is the consumers’ preference for high-quality consumption.
*Q₁*	Is the output of green agricultural products.
*P₁*	Is the price of green agricultural products.
*Rm₂*	Income of universities that engage in the negative R&D of green agricultural products, *Rm₂* = P₂*Q₂ (*Rm₂* <* Rm₁).*
*Q₂*	Is the output of ordinary agricultural products.
*P₂*	Price of ordinary agricultural products.
α*Cg₁	Total cost of government subsidies to universities when the government adopts a subsidy strategy.
β*Cg₂	Total cost of advertising to consumers when the government adopts a publicity strategy.
*α*	Subsidy coefficient for acquiring research equipment.
*β*	Advertising coefficient for consumers who choose to upgrade their consumption.
*Rg*	Government benefits from agricultural sustainability when the willingness for greenresearch and development in universities is high.
*Cg₃*	When the government refuses incentive policies, inaction leads to increased management costs (*Cg₃* > α*Cg₁ + β*Cg₂).
*Cf₁*	The R&D costs prior to universities’ active engagement in green agricultural product research.
*Cf₂*	The R&D costs prior to universities’ active engagement in green agricultural product development.(Cf₂ > Cf₁).
*G*	The negative impact on universities engaged inresearch and development of green agricultural products due to the absence of government subsidies, *G* = α*Cg₁.
*Sc*	Consumer search costs for green agricultural products.
*Rm₁*	The purchase cost is consumers will pay when choosing to upgrade their consumption.
*Rm₂*	The purchase cost is consumers will pay for choosing traditional consumption (*Rm₁* > *Rm₂*).
*S*	The social recognition consumers gain when government propaganda forms social norms (*S* > *Rm₁-Rm₂*).

**Table 2:** Tripartite game payoff matrix.

		Consumers	Government	
			Implementing incentive policies (*x*)	Reject Incentive Policy (1-*x*)
Universities	Choose to engage in the R&D of green agricultural products (*y*)	Upgrading consumption (*z*)	Rg-α*Cg₁-β*Cg₂, i*P₁*Q₁ + α*Cg₁-Cf₂, S-i*P₁*Q₁	*-Cg₃,* i*P₁*Q₁-Cf₂, -i*P₁*Q₁
		Traditional consumption (1-*z*)	-α*Cg₁-β*Cg₂, i*P₁*Q₁ + α*Cg₁ - Cf₂, -P₂*Q₂	*-Cg₃,* i*P₁*Q₁-Cf₂, -P₂*Q₂
	Refusal to engage in the R&D of green agricultural products (1-*y*)	Upgrading consumption (*z*)	-α*Cg₁-β*Cg₂, P₂*Q₂-Cf₁-α*Cg₁, S-P₂*Q₂ − Sc	*-Cg₃,* P₂*Q₂-Cf₁, -i*P₁*Q₁-Sc
		Traditional consumption (1-*z*)	-α*Cg₁-β*Cg₂, P₂*Q₂-Cf₁-α*Cg₁, -P₂*Q₂	*-Cg₃,* P₂*Q₂-Cf₁, -P₂*Q₂

### 3.2. Expectations and replication dynamics equations for different participants

Using the established evolutionary game framework and its corresponding payoff matrix, we can calculate the expected utilities and average payoffs for all stakeholders (government, universities, and consumers) across various strategy combinations. The detailed calculations proceed as follows:

The expected benefits of the government adopting and rejecting incentive policies are denoted as V11and V12, respectively, with the average benefit being V1, which can be expressed using formulas (1), (2), and (3).


$$\begin{array}{cc} V\text{11}= & \;(Rg-\alpha *{Cg}_{\text{1}}-\beta *{Cg}_{\text{2}})*y*z+(-\alpha *{Cg}_{\text{1}}-\beta *{Cg}_{\text{2}})*y*(\text{1}-z)+(-\alpha *{Cg}_{\text{1}}-\beta {Cg}_{\text{2}})*(\text{1}-y)*z\\ & +(-\alpha {Cg}_{\text{1}}-\beta {Cg}_{\text{2}})*(\text{1}-y)*(\text{1}-z) \end{array}$$ (1)

$$V\text{1}\text{2}=(-{Cg}_{\text{3}})*y*z+(-{Cg}_{\text{3}})*y*(\text{1}-z)+(-{Cg}_{\text{3}})*(\text{1}-y)*z+(-{Cg}_{\text{3}})*(\text{1}-y)*(\text{1}-z)$$ (2)

$$V\text{1}=x*V\text{1}\text{1}+(\text{1}-x)*V\text{1}\text{2}={Cg}_{\text{3}}*x-{Cg}_{\text{3}}-\alpha *{Cg}_{\text{1}}-\beta *{Cg}_{\text{2}}*x+Rg*x*y*z$$ (3)

The expected benefits of universities actively developing or passively developing green agricultural products are denoted as V21 and V22 respectively, with the average benefit being V2. These can be expressed using Equations (4), (5), and (6).


$$\begin{array}{cc} V\text{2}\text{1}= & \;(i*P_{\text{1}}*Q_{\text{1}}+\alpha *{Cg}_{\text{1}}-{Cf}_{\text{2}})*x*z+(i*P_{\text{1}}*Q_{\text{1}}+\alpha *{Cg}_{\text{1}}-{Cf}_{\text{2}})*x*(\text{1}-z)\\ & +(i*P_{\text{1}}*Q_{\text{1}}-{Cf}_{\text{2}})*(\text{1}-x)*z+(i*P_{\text{1}}*Q_{\text{1}}-{Cf}_{\text{2}})*(\text{1}-x)*(\text{1}-z) \end{array}$$ (4)

$$\begin{array}{cc} V\text{2}\text{2}= & \;(P_{\text{2}}*Q_{\text{2}}-{Cf}_{\text{1}}-\alpha *{Cg}_{\text{1}})*x*z+(P_{\text{2}}*Q_{\text{2}}-{Cf}_{\text{1}}-\alpha *{Cg}_{\text{1}})*x*(\text{1}-z)\\ & +(P_{\text{2}}*Q_{\text{2}}-{Cf}_{\text{1}})*(\text{1}-x)*z+(P_{\text{2}}*Q_{\text{2}}-{Cf}_{\text{1}})*(\text{1}-x)*(\text{1}-z) \end{array}$$ (5)

$$\begin{array}{cc} V\text{2}= & \;y*V\text{2}\text{1}+(\text{1}-y)*V\text{2}\text{2}=P_{\text{2}}*Q_{\text{2}}+{Cf}_{\text{1}}+{Cf}_{\text{1}}*y-{Cf}_{\text{2}}*y-P_{\text{2}}*Q_{\text{2}}*y-\alpha *{Cg}_{\text{1}}*x\\ & +P_{\text{1}}*Q_{\text{1}}*i*y+\text{2}*\alpha *{Cg}_{\text{1}}*x*y \end{array}$$ (6)

The expected benefits of consumers choosing and rejecting consumption upgrades are represented by V31 and V32, respectively, with the average benefit being V3, which can be expressed using formulas (7), (8), and (9).


$$\begin{array}{cc} V\text{3}\text{1}= & \;(S-i*P_{\text{1}}*Q_{\text{1}})*x*y+(S-P_{\text{2}}*Q_{\text{2}}-Sc)*x*(\text{1}-y)+(-i*P_{\text{1}}*Q_{\text{1}})*(\text{1}-x)*y\\ & +(-i*P_{\text{1}}*Q_{\text{1}}-Sc)*(\text{1}-x)*(\text{1}-y) \end{array}$$ (7)

$$V\text{3}\text{2}=(-P_{\text{2}}*Q_{\text{2}})*x*y+(-P_{\text{2}}*Q_{\text{2}})*x*(\text{1}-y)+(-P_{\text{2}}*Q_{\text{2}})*(\text{1}-x)*y+(-P_{\text{2}}*Q_{\text{2}})*(\text{1}-x)*(\text{1}-y)$$ (8)

$$\begin{array}{cc} V\text{3}= & \;z*V\text{3}\text{1}+(\text{1}-z)*V\text{3}\text{2}=z*(x*(y-\text{1})*(Sc-S\;+P_{\text{2}}*Q_{\text{2}}))\\ & -(Sc+P_{\text{1}}*Q_{\text{1}}*i)*(x-\text{1})*(y-\text{1})+x*y*(S-P_{\text{1}}*Q_{\text{1}}*i)+P_{\text{1}}*Q_{\text{1}}*i*y*(x-\text{1}))\\ & -(z-\text{1})*(P_{\text{2}}*Q_{\text{2}}*x*(y-\text{1})+P_{\text{2}}*Q_{\text{2}}*y*(x-\text{1})\\ & -P_{\text{2}}*Q_{\text{2}}*(x-\text{1})*(y-\text{1})-P_{\text{2}}*Q_{\text{2}}*x*y) \end{array}$$ (9)

According to evolutionary game theory, the replication dynamics equations for the government, universities, and consumers can be calculated based on their expected benefits, as shown below. Equations (10), (11), and (12) are as follows:


$$F(x)\;=\;dx/dt\;+\;x(V\text{1}\text{1}\;-\;V\text{1})\;=\;x(\text{1}\;-\;x)(V\text{1}\text{1}-\;V\text{1}\text{2})=-x*(x-\text{1})*({Cg}_{\text{3}}-\alpha *{Cg}_{\text{1}}-{Cg}_{\text{2}}*\beta +Rg*y*z)$$ (10)

$$F(y)\;=\;dy/dt\;+\;y(V\text{2}\text{1}-\;V\text{2}\text{2})\;=\;y(\text{1}\;-\;y)(V\text{2}\text{1}-\;V\text{2}\text{2})=\;-y*(y-\text{1})*({Cf}_{\text{1}}-{Cf}_{\text{2}}-P_{\text{2}}*Q_{\text{2}}+\;P_{\text{1}}*Q_{\text{1}}*i+\text{2}*\alpha *{Cg}_{\text{1}}*x)$$ (11)

$$\begin{array}{cc} F(z) & \;=\;dz/dt\;=\;z(V\text{3}\text{1}\;-\;V\text{3})\;=\;z(\text{1}\;-\;z)(V\text{3}\text{1}\;-\;V\text{3}\text{2})\\ & \;=-z*(z-\text{1})*(P_{\text{2}}*Q_{\text{2}}-Sc+S*x+Sc*y-\;P_{\text{1}}*Q_{\text{1}}*i-P_{\text{2}}*Q_{\text{2}}*x\\ & \;\;\;\;\;+\;P_{\text{1}}*Q_{\text{1}}*i*x+P_{\text{2}}*Q_{\text{2}}*x*y-\;P_{\text{1}}*Q_{\text{1}}*i*x*y) \end{array}$$ (12)

### 3.3. Analysis of local stable strategies for participants

When the replicator dynamic equation is zero (F(x)=0, F(y)=0, F(z)=0), the strategy variables (x, y, z) achieve a stable state with no further temporal evolution. At this equilibrium point, all agents have implemented their optimal strategies. Consequently, we now analyse the stability conditions for each stakeholder group’s strategy---government, universities, and consumers---as detailed below:

For the government, according to Equation (10), when F(*x*) = 0, *x* = 0 or **x* *= 1, the following conclusions can be drawn:

1. When ${\text{Cg}}_{\text{3}}+\text{Rg}*\text{y}*\text{z}>{\text{Cg}}_{\text{1}}*\alpha +{\text{Cg}}_{\text{2}}*\beta$, F(x) > 0. At this point, y = 1, meaning the government will ultimately choose an incentive policy.
2. When ${\text{Cg}}_{\text{3}}+\text{Rg}*\text{y}*\text{z}<{\text{Cg}}_{\text{1}}*\alpha +{\text{Cg}}_{\text{2}}*\beta$, F(*x*) < 0. At this point, **y* *= 0, meaning the government will ultimately reject incentive policies.

For universities, according to Equation (11), when F(*y*) = 0, **y* *= 0 or *y* = 1, the following conclusions can be drawn:

1. When ${Cf}_{\text{1}}+P_{\text{1}}*Q_{\text{1}}*i+\text{2}*{Cg}_{\text{1}}*\alpha *x>{Cf}_{\text{2}}+P_{\text{2}}*Q_{\text{2}}$, F(*y*) > 0. At this point, **y* *= 1, meaning that the benefits of choosing to R&D green agricultural products for universities are greater than the benefits of refusing to R&D green agricultural products. Therefore, these universities will ultimately choose to R&D green agricultural products.
2. When ${Cf}_{\text{1}}+P_{\text{1}}*Q_{\text{1}}*i+\text{2}*{Cg}_{\text{1}}*\alpha *x={Cf}_{\text{2}}+P_{\text{2}}*Q_{\text{2}}$, F(*y*) = 0. At this point, the benefits of the universities choosing or rejecting the R&D of green agricultural products are the same, and all values are in an evolutionary stable state.
3. When $\;{Cf}_{\text{1}}+P_{\text{1}}*Q_{\text{1}}*i+\text{2}*{Cg}_{\text{1}}*\alpha *x<{Cf}_{\text{2}}+P_{\text{2}}*Q_{\text{2}}$, F(*y*) < 0. At this point, **y* *= 0, meaning that the benefits of rejecting the R&D of green agricultural products are greater than those of choosing to R&D green agricultural products. Therefore, these universities will ultimately reject the R&D of green agricultural products.

For consumers, from Equation (12), when F(*z*) = 0, **z* *= 0 or **z* *= 1, the following conclusions can be drawn:

1. When $P_{\text{2}}*Q_{\text{2}}+S*x+Sc*y+P_{\text{1}}*Q_{\text{1}}*i*x+P_{\text{2}}*Q_{\text{2}}*x*y>Sc+P_{\text{1}}*Q_{\text{1}}*i+P_{\text{2}}*Q_{\text{2}}*x+P_{\text{1}}*Q_{\text{1}}*i*y$, F(*z*) > 0. At this point, **z* *= 1, meaning that consumers will ultimately choose to upgrade their consumption.
2. When $P_{\text{2}}*Q_{\text{2}}+S*x+Sc*y+P_{\text{1}}*Q_{\text{1}}*i*x+P_{\text{2}}*Q_{\text{2}}*x*y<Sc+P_{\text{1}}*Q_{\text{1}}*i+P_{\text{2}}*Q_{\text{2}}*x+P_{\text{1}}*Q_{\text{1}}*i*y$, F(*z*) < 0. At this point, **z* *= 0, meaning that consumers will ultimately refuse to upgrade their consumption.

### 3.4. Stability analysis of the evolutionary system

Under conditions of information asymmetry, the evolutionarily stable strategy (ESS) in asymmetric games must be a pure strategy. Setting the replicator dynamic Equations (10)-(12) equal to zero yields eight pure-strategy equilibrium points: E₁(0,0,0), E₂(1,0,0), E₃(0,1,0), E₄(0,0,1), E₅(1,1,0), E₆(0,1,1), E₇(1,0,1), and E₈(1,1,1). However, directly identifying the optimal equilibrium point among these candidates is often challenging. We therefore apply Lyapunov’s stability criterion by analyzing the Jacobian matrix to evaluate the asymptotic stability of these equilibrium points. According to this method, an equilibrium achieves asymptotic stability if and only if all eigenvalues of the corresponding Jacobian matrix possess negative real parts. We therefore construct the Jacobian matrix by computing the first-order partial derivatives of F(x), F(y), and F(z) with respect to x, y, and z, respectively. The eigenvalues of this matrix then allow us to analyze evolutionary stability trends among the government, universities, and consumers, as presented in Equation (13):


$$\;j=\left(\;\begin{array}{ccc} J_{\text{1}\text{1}} & J_{\text{1}\text{2}} & J_{\text{1}\text{3}}\\ J_{\text{2}\text{1}} & J_{\text{2}\text{2}} & J_{\text{2}\text{3}}\\ J_{\text{3}\text{1}} & J_{\text{3}\text{2}} & J_{\text{3}\text{3}} \end{array}\;\right)=\left(\;\begin{array}{ccc} \frac{\partial F(x)}{\partial x} & \frac{\partial F(x)}{\partial y} & \frac{\partial F(x)}{\partial z}\\ \frac{\partial F(y)}{\partial x} & \frac{\partial F(y)}{\partial y} & \frac{\partial F(y)}{\partial z}\\ \frac{\partial F(z)}{\partial x} & \frac{\partial F(z)}{\partial y} & \frac{\partial F(z)}{\partial z} \end{array}\;\right)$$ (13)

$$J_{\text{1}\text{1}}=\;-\;x*({Cg}_{\text{3}}-\;{Cg}_{\text{1}}*\alpha -\;{Cg}_{\text{2}}*\beta +\;Rg*y*z)\;-\;(x\;-\;\text{1})*({Cg}_{\text{3}}-\;{Cg}_{\text{1}}*\alpha \;-\;{Cg}_{\text{2}}*\beta \;+\;Rg*y*z)$$

$$\begin{array}{c} J_{\text{1}\text{2}}=-Rg*x*z*(x\;-\;\text{1})\\ J_{\text{1}\text{3}}=-Rg*x*y*(x\;-\;\text{1}) \end{array}$$

$$J_{\text{2}\text{1}}=-\text{2}*Cg\text{1}*\alpha *y*(y\;-\;\text{1})$$

$$\begin{array}{cc} J_{\text{2}\text{2}}= & -y*(Cf\text{1}-Cf\text{2}-P\text{2}*Q\text{2}+P\text{1}*Q\text{1}*i+\text{2}*Cg\text{1}*\alpha *x)-(y\;-\text{1})*(Cf\text{1}-Cf\text{2}\;\\ & -P\text{2}*Q\text{2}\;+\;P\text{1}*Q\text{1}*i+\text{2}*Cg\text{1}*\alpha *x) \end{array}$$

$$J_{\text{2}\text{3}}=\text{0}$$

$$J_{\text{3}\text{1}}=\;-z*(z\;-\;\text{1})*(S\;-P_{\text{2}}*Q_{\text{2}}+\;\;P_{\text{1}}*Q_{\text{1}}*i\;+\;P_{\text{2}}*Q_{\text{2}}*y\;-\;P_{\text{1}}*Q_{\text{1}}*i*y)$$

$$J_{\text{3}\text{2}}=-\;z*(P\text{2}*Q\text{2}\;-\;Sc\;+\;S*x\;+\;Sc*y\;-\;P\text{1}*Q\text{1}*i\;-\;P\text{2}*Q\text{2}*x\;+\;P\text{1}\;*Q\text{1}*i*x\;+\;P\text{2}*Q\text{2}*x*y$$

$$\begin{array}{cc} J_{\text{3}\text{3}}= & \;-\;P\text{1}*Q\text{1}*i*x*y)-(z-\text{1})*(P\text{2}*Q\text{2}-Sc+S*x+Sc*y-P\text{1}*Q\text{1}*i-P\text{2}*Q\text{2}*x+P\text{1}*Q\text{1}*i*x\\ & +P\text{2}*Q\text{2}*x*y-P\text{1}*Q\text{1}*i*x*y) \end{array}$$

Following Lyapunov’s indirect method, the eigenvalues derived from the Jacobian matrix provide definitive criteria for assessing the stability of evolutionary dynamic systems. Specifically, at any equilibrium point: complete positivity of all eigenvalues’ real parts indicates instability; exclusively negative real parts characterise an evolutionarily stable state; mixed signs in the real parts identify a saddle point. Through substitution of the eight equilibrium points into Equation (13), we calculated the corresponding eigenvalues and recorded their signs in Table 3. In this table, the notations “+”, “-”, and “s” denote eigenvalues with positive, negative, and indeterminate signs, respectively.

**Table 3:** Equilibrium points and eigenvalues.

Equilibrium Points	Jacobian Eigenvalue	Actual Situation	Stability
	*λ₁, λ₂, λ₃*		
E₁= (0, 0, 0)	$P_{\text{2}}*Q_{\text{2}}-Sc-P_{\text{1}}*Q_{\text{1}}*i$, ${Cg}_{\text{3}}-{Cg}_{\text{1}}*\alpha -{Cg}_{\text{2}}*\beta$, ${Cf}_{\text{1}}-{Cf}_{\text{2}}-P_{\text{2}}*Q_{\text{2}}+P_{\text{1}}*Q_{\text{1}}*i$	(-, + ,s)	Saddle point
E₂=(1,0,0)	S−Sc, ${Cg}_{\text{1}}*\alpha -{Cg}_{\text{3}}+{Cg}_{\text{2}}*\beta$, ${Cf}_{\text{1}}-{Cf}_{\text{2}}-P_{\text{2}}*Q_{\text{2}}+\text{2}*{Cg}_{\text{1}}*\alpha +P_{\text{1}}*Q_{\text{1}}*i$	(s,-,s)	Saddle point or stable point
E₃= (0, 1, 0)	$P_{\text{2}}*Q_{\text{2}}-P_{\text{1}}*Q_{\text{1}}*i$ ${Cg}_{\text{3}}-{Cg}_{\text{1}}*\alpha -{Cg}_{\text{2}}*\beta$, ${Cf}_{\text{2}}-{Cf}_{\text{1}}+P_{\text{2}}*Q_{\text{2}}-P_{\text{1}}*Q_{\text{1}}*i$	(-, + ,-)	Saddle point
E₄=(0,0,1)	$Sc-P_{\text{2}}*Q_{\text{2}}+P_{\text{1}}*Q_{\text{1}}*i$, ${Cg}_{\text{3}}-{Cg}_{\text{1}}*\alpha -{Cg}_{\text{2}}*\beta$, ${Cf}_{\text{1}}-{Cf}_{\text{2}}-P_{\text{2}}*Q_{\text{2}}+P_{\text{1}}*Q_{\text{1}}*i$	(+,s,s)	Saddle point
E₅=(1,1,0)	${Cg}_{\text{1}}*\alpha -{Cg}_{\text{3}}+{Cg}_{\text{2}}*\beta$, $S+P_{\text{2}}*Q_{\text{2}}-P_{\text{1}}*Q_{\text{1}}*i$, ${Cf}_{\text{2}}-{Cf}_{\text{1}}+P_{\text{2}}*Q_{\text{2}}-\text{2}*{Cg}_{\text{1}}*\alpha -P_{\text{1}}*Q_{\text{1}}*i$	(-,-,s)	Saddle point or stable point
E₆=(0,1,1)	$P_{\text{1}}*Q_{\text{1}}*i-P_{\text{2}}*Q_{\text{2}}$, ${Cf}_{\text{2}}-{Cf}_{\text{1}}+P_{\text{2}}*Q_{\text{2}}-P_{\text{1}}*Q_{\text{1}}*i$, ${Cg}_{\text{3}}+Rg-{Cg}_{\text{1}}*\alpha -{Cg}_{\text{2}}*\beta$	(+,s,+)	Saddle point
E₇=(1,0,1)	Sc−S, ${Cg}_{\text{1}}*\alpha -{Cg}_{\text{3}}+{Cg}_{\text{2}}*\beta$, ${Cf}_{\text{1}}-{Cf}_{\text{2}}-P_{\text{2}}*Q_{\text{2}}+\text{2}*{Cg}_{\text{1}}*\alpha +P_{\text{1}}*Q_{\text{1}}*i$	(s, s, -)	Saddle point or stable point
E₈=(1,1,1)	$P_{\text{1}}*Q_{\text{1}}*i-P_{\text{2}}*Q_{\text{2}}-S$, ${Cg}_{\text{1}}*\alpha -Rg-{Cg}_{\text{3}}+{Cg}_{\text{2}}*\beta$, ${Cf}_{\text{2}}-{Cf}_{\text{1}}+P_{\text{2}}*Q_{\text{2}}-\text{2}*{Cg}_{\text{3}}*\alpha -P_{\text{1}}*Q_{\text{1}}*i$	(-,-,s)	Saddle point

Based on our established assumptions and the Lyapunov indirect method, we conclude that four equilibrium points---E₁(0,0,0), E₃(0,1,0), E₄(0,0,1), and E₆(0,1,1)---cannot be evolutionarily stable strategies (ESS). This indicates scenarios where either one stakeholder’s interests are maximised at the expense of the other two, or where all three parties’ interests remain suboptimal. We therefore exclude these four points from further analysis. Aligned with this study’s value orientation, we focus on scenarios where universities actively engage in digital transformation, stimulated by government incentive policies and consumer demand for upgraded consumption. Consequently, we also exclude cases E₂(1,0,0) and E₇(1,0,1), and focus our analysis exclusively on the potentially stable states E₅(1,1,0) and E₈(1,1,1).

### 3.5. Scenario analysis of the evolutionary system

#### 3.5.1. Data sources.

In the current era, green production technologies are exerting a powerful influence, providing critical support conditions for the green and sustainable development of agriculture. From resource utilization, industrial integration to production management and operational decision-making, these technologies are comprehensively reshaping the development landscape of agriculture [28]. W. Schlenker and M. Roberts [29] noted that corn and soybean yields decline sharply once temperatures reach a certain threshold, and this non-linear relationship indicates that climate change significantly impacts crop yields. However, environmental instability may lead to fluctuations in agricultural product quality. This increases the difficulty of tracking and managing the stability of agricultural product quality during the green transformation process, which is unfavourable for the green transformation efforts of universities. Additionally, other universities may lack the strong research team support available to the Zhejiang Cangnan Blue Crab Science and Technology Backyard, resulting in relatively limited professional knowledge and experience among researchers. This may result in lower technical levels in production technology, quality testing, disease prevention and control, and an inability to effectively ensure the quality and safety of agricultural products [30], which is unfavourable for the implementation of green agricultural product R&D processes. Therefore, this study takes the green agricultural product research of the Zhejiang Cangnan Blue Crab Science and Technology Backyard as an example. On the one hand, blue crabs are a high-end seafood product with tender meat and rich nutritional value, enjoying high market demand and prices. In both domestic and international markets, blue crabs command relatively high prices, generating significant economic benefits for farmers. On the other hand, traditional blue crab farming is largely extensive, lacking scientific management and technical guidance. Low stocking densities and unstable yields result in slow growth rates and inconsistent product quality. Therefore, the Cangnan Blue Crab universities in Zhejiang Province adopted a “mangrove planting-ecological farming coupling” model, integrating the mangrove ecosystem with blue crab farming to create an optimal growth environment for blue crabs. This model not only improves the quality of blue crabs but also achieves a win-win outcome of ecological and economic benefits, aligning closely with the current national food safety strategy and requirements for green agricultural development.

This study selects Wenzhou City in Zhejiang Province as the research area for three key reasons. First, Wenzhou has established a comprehensive policy framework and technical application system for green agriculture. Second, the region demonstrates advanced levels of agricultural digitalisation and smart farming, with technologies such as the Agricultural Internet of Things and intelligent equipment being widely implemented. This holds significant reference value for China’s agricultural green R&D. As a pioneer province in China’s digital reform, Zhejiang has consistently led the way in the green development of digital agriculture. The Zhejiang Cangnan Blue Crab Science and Technology Backyard has significant advantages in the R&D of green agricultural products. Relying on a team of eight experts led by doctoral students from Zhejiang Ocean university, it has been deeply involved in mangrove planting and ecological farming. The monthly “industry-academia-research” practical activities of graduate students not only inject innovative technologies into blue crab ecological farming but also cultivate practical talents, thereby supporting a unique ecological farming model. Through “mangrove planting - ecological farming coupling”, it achieves a win-win outcome for ecology and economy. Mangroves purify water quality to create an optimal environment for blue crabs, while blue crabs and other organisms in turn benefit the mangrove ecosystem. Additionally, ecological restoration of tidal flats and stock enhancement through release further enhance ecosystem stability and productivity. Based on this, the quality inspection laboratory, breeding system, and seedling base planned and constructed by the universities strictly control the quality of blue crabs, while the registration of the “mangrove blue crab” brand has enhanced the product’s market competitiveness; Additionally, the Cangnan County Government has placed high priority on this initiative, issuing multiple policy documents covering financial support, tax incentives, and land use guarantees to comprehensively support the development of the universities. It actively promotes deep cooperation between the village, universities, and enterprises, guiding resources to converge towards the village. Enterprises have also actively participated, investing significant funds to expand mangrove planting scale, restore bare tidal flats, and conduct ecological aquaculture. Their strong market operation capabilities have effectively driven the sales and promotion of green agricultural products. In terms of actual achievements, the universities have achieved remarkable results. Through the efforts of the expert team, the mangrove planting area in Yanpu Bay has expanded from its initial stage to nearly 2,000 acres, with ecological functions becoming increasingly prominent. The water quality in the surrounding waters has significantly improved; the diversity and abundance of benthic organisms have greatly increased, and the production of blue crabs has steadily increased with noticeable improvements in quality. The market price of blue crabs is higher than that of ordinary blue crabs, achieving a balance between ecological protection and economic benefits. Its sustainable development model has set a benchmark for the industry, serving as a demonstration site to provide valuable experience for the transformation of traditional aquaculture, driving the development of green agriculture on a larger scale, and leading the agricultural industry towards a sustainable future.

The initial parameter values for this investigation were sourced from six distinct origins: (1) Field investigation. Through research on the Zhejiang Cangnan Blue Crab Science and Technology Backyard, we found that it is a science and technology service institution established in collaboration with Zhejiang Ocean University, dedicated to promoting the ecological transformation and sustainable development of the blue crab industry in Cangnan and surrounding areas. By developing a mangrove ecological farming model and creating the “Jiwei Blue Crab” brand, the Zhejiang Cangnan Blue Crab Science and Technology Backyard has significantly improved production efficiency and ensured food safety. Its core work includes mangrove ecological restoration and protection, the promotion of ecological farming technologies, and collaborative innovation between academia, research, and industry.Currently, the Science and Technology Backyard, in collaboration with an enterprise, has completed the ecological restoration of 300 mu of tidal flats. Through stocking and natural breeding, it has enriched benthic resources such as blue crabs and mudskippers. This has established a “mangrove planting-ecological aquaculture coupling” symbiotic model, providing a demonstration for upgrading traditional aquaculture practices across the province.Under the green farming method, the quality of blue crabs from the base’s pen culture has improved significantly, commanding an average selling price of 190 yuan per kilogram. In contrast, blue crabs farmed by ordinary farmers may be subject to certain artificial interventions and constraints in their growing environment, and the feed used is more conventional. Consequently, the flesh texture and taste of the resulting crabs are relatively ordinary, and their size and morphology are less uniform. As a result, their price typically falls into the low-to-medium range, averaging 100 yuan per kilogram.

(2) Measurement and CalculationBased: on local market conditions, we conducted a detailed calculation of the production costs for blue crab farming. Cultivating high-quality blue crabs in base pens requires the construction of corresponding farming facilities. These include standardised aquaculture ponds, water purification equipment, aeration systems, drainage systems, and anti-escape facilities. Constructing a standardised 1,000-square-metre aquaculture pond, encompassing tasks such as pond levelling, anti-seepage treatment, installation of inlet/outlet pipes, and fitting of aeration equipment, may require an investment of approximately 50,000 yuan. If more advanced farming equipment is adopted, such as recirculating water treatment systems or intelligent temperature control systems used in industrial aquaculture, the costs will be higher. The cost per square metre could reach 100–200 yuan. Furthermore, feed constitutes one of the primary costs in the farming process. However, feed costs can vary depending on factors such as stocking density and the farming cycle. Therefore, for the purposes of this analysis, we assume that feed costs are consistent between conventional and green farming methods. Thus, the cost for conventional farmers using standardised facilities is 50,000 yuan, whereas the cost for the university cultivating high-quality blue crabs is 200,000 yuan.
(3) Related Documents: Government special subsidy policies provide crucial support for the modernisation of agricultural science and technology. A typical policy basis is the “Notice of the Zhejiang Provincial Department of Finance and the Zhejiang Provincial Department of Science and Technology on Issuing the Measures for the Administration of Special Funds for Science and Technology Development in Zhejiang Province” (hereinafter referred to as the “Notice”). The Notice specifies differentiated subsidy standards for agricultural science and technology projects led by different entities. For projects led by enterprises in the agricultural sector or by enterprises in mountainous and island counties, the subsidy ratio does not exceed 40%. For industrialisation cooperation projects led by universities or research institutes, the subsidy ratio does not exceed 50%. Based on the policy orientation and the rationale of the research design, this study sets the government’s initial subsidy coefficient at 0.4. Regarding the verification of indirect costs, the Notice stipulates that indirect costs are determined as a certain percentage of the direct costs after deducting equipment purchase expenses. Specifically, for portions up to 5 million yuan, the rate is set at 30%; for portions between 5 million and 10 million yuan, at 25%; and for portions exceeding 10 million yuan, at 20%. Government expenditure on promoting green consumption concepts through television advertisements falls within this category of indirect costs. Considering both policy requirements and the applicability of research data, this study selects 30% as the government publicity coefficient. The Notice also specifies that the maximum provincial financial subsidy for a single agricultural science and technology project shall not exceed 10 million yuan, with projects demonstrating outstanding performance eligible for rolling support. For major science and technology projects implemented by provincial innovation consortia, the linked subsidies from provincial, municipal, and county levels can reach up to 30 million yuan. Integrating the aforementioned policy ceilings with the practical needs of the research, this study sets the government subsidy amounts as follows: the government’s research and development subsidy to the university (α*Cg₁) is set at 3 million yuan, and the publicity subsidy (β*Cg₂) at 2.5 million yuan. Given that Cg₃ needs to be higher than the sum of the previous two subsidies, Cg₃ is ultimately set at 6 million yuan.
(4) Questionnaire: To verify the degree of consumer preference for high-quality agricultural products, this study conducted a specialised questionnaire survey (sample size n = 105). The survey results indicate a consumer preference coefficient for high-quality agricultural products of 0.7. Among the 105 respondents, 73 explicitly expressed support for quality-oriented agricultural product consumption behaviour. This further provides data support for the demand-side assumptions of the research.
(5) Classical Literature: Conceptually, consumer search cost represents the opportunity cost of the time and effort consumers expend during the product search process. As established by Hong and Shum in “Using Price Distributions to Estimate Search Costs” [31], these costs can be quantified as the implicit labour costs borne by consumers during the search period.
(6) Expert Consultation: Behavioural economics experts estimated the opportunity cost of non-green production and the utility consumers derive from quality consumption. This study will utilise annualised data for a 1,000-square-metre farming unit. For intensive pond culture, based on a standard stocking density of 2,000 seedlings per mu, a 1,000-square-metre area (approximately 1.5 mu) can support the farming of 3,000 blue crabs. The individual weight is assumed to be 500 grams (within the normal range of 200–500 grams). All parameters are simplified and evaluated as shown in Table 4.

**Table 4:** Variable data assignments.

Main Variable	Variable	Variable	Value	Unit	Data Source
Government	*α*	Government R&D subsidy coefficient	40	%	Related Documents
	*β*	Government Promotion Coefficient	30	%	Related Documents
	α*Cg₁	R&D Subsidy Cost	8	Ten thousand yuan	Related Documents
	β*Cg₂	Publicity Cost	6	Ten thousand yuan	Related Documents
	*Rg*	When the universities have a high willingness to research, develop, and produce green agricultural products, the government benefits from sustainable agricultural development	10	Ten thousand	Expert Estimates
	*Cg₃*	When the government rejects policy incentives, inaction leads to increased management costs (*Cg₃* > α*Cg₁ + β*Cg₂).	15	Ten thousand yuan	Related Documents
Universities	*Rm₁*	Revenue generated by universities actively engaged in R&D, *Rm₁* = i*P₁*Q₁	20	Ten thousand yuan	Expert Estimates
	*i*	represents consumers’ premium consumption preferences	70	%	Questionnaire
	*Q₁*	Output of high-quality green-farmed blue crabs	1500	Kilogram	Expert Estimates
	*P₁*	Price of high-quality green-farmed blue crabs	190	Yuan per kilogram	Field investigation
	*Rm₂*	Income from conventionally farmed blue crabs, Rm₂ = P₂*Q₂ (*Rm₂ < Rm₁*)	15	Ten thousand yuan	Expert Estimates
	*Q₂*	Is the yield of farmed blue crabs	1500	Kilogram	Expert Estimates
	*P₂*	Is the price of commonly farmed blue crabs	100	Yuan per kilogram	Field investigation
	*Cf₁*	Production costs for green farming	20	Ten thousand yuan	Measurement and CalculationBased
	*Cf₂*	Production costs of conventional aquaculture (*Cf₁ *>* Cf₂*)	5	Ten thousand yuan	Measurement and CalculationBased
	*G*	The loss incurred by universities without government subsidies, *G* = α*Cg₁	10	Ten thousand	Expert Estimates
Consumers	*Sc*	Cost to consumers of searching for green agricultural products	0.045	Ten thousand yuan	Classical Literature
	*Rm₁*	Purchase cost incurred by consumers who upgrade their consumption	20	Ten thousand yuan	Expert Estimates
	*Rm₂*	Purchase cost incurred by consumers choosing traditional consumption	15	Ten thousand yuan	Expert Estimates
	*S*	Social recognition gained by consumers when government promotion establishes social norms (*S *> *Rm₁- Rm₂*)	10	Ten thousand yuan	Expert Estimates

#### 3.5.2. Scenario analysis.

Scenario 1: The strategic combination (1,1,0) constitutes an evolutionarily stable strategy (ESS). As shown in Table 2, the conditions specified by inequality set (14) are satisfied. This confirms that the government’s incentive policies yield greater benefits for universities engaged in green agricultural research and development. However, the impact of these publicity efforts on consumers is relatively weak, resulting in a failure to foster widespread upgraded consumption behaviour.


$$\alpha *{Cg}_{\text{1}}+\beta *{Cg}_{\text{2}}<{Cg}_{\text{3}}$$ (14)

$$S+P_{\text{2}}*Q_{\text{2}}<P_{\text{1}}*Q_{\text{1}}*i$$

**Fig 3:**
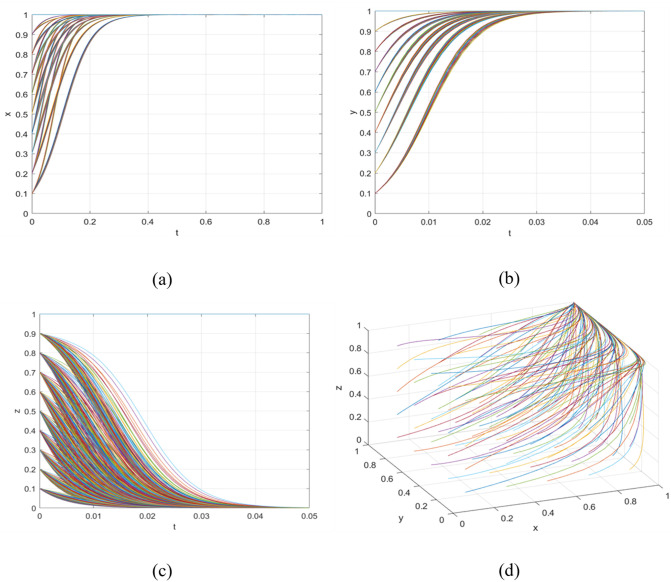
The sub-figures illustrate the individual and collective strategic paths: (a) depicts the government’s strategic path over time; (b) shows the corresponding path for universities; (c) illustrates the consumer strategy evolution; while (d) presents the combined behavioural trends of all three groups.

Scenario 1 indicates that the government’s publicity efforts towards consumers are insufficient, failing to incentivise them to actively choose premium consumption. As can be seen from Fig 3, the government has successfully guided universities towards sustainable R&D through subsidies and publicity. This governance objective—promoting green transformation on the supply side—has been partially achieved. Supported by these policies, universities have internalised sustainable R&D as a rational choice. Even if the consumption side does not upgrade immediately, their R&D activities may still yield returns through government procurement, special projects, or long-term market cultivation. However, consumers have not yet formed widespread premium consumption behaviour. This is due to factors such as excessive product premiums, insufficient awareness of sustainable products, or the strong inertia of existing consumption habits. This may be because government publicity has failed to effectively communicate value signals, or because the green products supplied to the market have not yet met the acceptance threshold of the mass market in terms of cost-effectiveness and availability. When the evolutionary system satisfies Scenario 1 constraints, it can reach a stable condition. To validate this finding, we utilised Matlab software to simulate the strategic evolution among government entities, universities, and consumers. Initial parameter values were configured to fulfil the conditions specified in inequality set (14). To elucidate evolutionary outcomes and account for regional policy differences, we modified variables controlling governmental policy intensity. Specifically, values of α = 0.6 and β = 0.7 were assigned, while maintaining all other parameters at their original values. Simulation results are presented in Fig 3. Subfigure Fig 3(a) demonstrates governmental adoption of incentive policies regardless of initial strategic positions; Subfigure Fig 3(b) indicates universities’ consistent choice to actively develop and manufacture green agricultural products, independent of starting conditions; Subfigure Fig 3(c) reveals consumers’ persistent selection of conventional consumption patterns, irrespective of initial preferences; Subfigure Fig 3(d) displays a three-dimensional representation of all parties’ evolutionary strategies, showing the system’s convergence to and stabilisation at point (1,1,0).

Scenario 2: The point (1,1,1) represents an evolutionarily stable strategy. According to Table 2, the inequality system (15) can be derived, which demonstrates that the governance costs of government inaction surpass those of implementing incentive policies. Consequently, the government will adopt incentive measures. For universities, the benefits of active engagement in green agricultural R&D outweigh those of passive involvement. Thus, universities opt for active R&D strategies. Simultaneously, the government’s publicity campaigns influence consumers towards consumption upgrade choices.


$$P_{\text{1}}*Q_{\text{1}}*i<S+P_{\text{2}}*Q_{\text{2}}$$ (15)

$$\alpha *{Cg}_{\text{1}}+\beta *{Cg}_{\text{2}}<Rg+{Cg}_{\text{3}}$$

**Fig 4:**
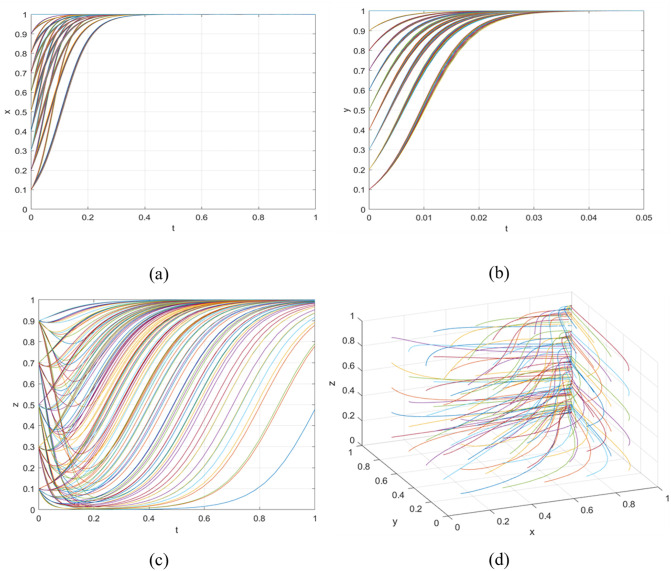
The sub-figures illustrate the individual and collective strategic paths: (a) depicts the government’s strategic pathway; (b) illustrates the behavioural path of universities; (c) shows the consumption strategy evolution; while (d) presents the integrated trends across all three groups.

Scenario 2 (1,1,1) demonstrates that government subsidies for R&D enable universities to partially offset losses encountered during active development of sustainable agricultural products. Consequently, universities show greater inclination towards active engagement in sustainable agricultural innovation. Government publicity initiatives enhance consumers’ preference for premium products, prompting voluntary adoption of upgraded consumption patterns. When the evolutionary system meets Scenario 2’s constraints, it achieves a stable equilibrium. Verification was conducted through Matlab simulations of strategic evolution among government entities, universities, and consumers. Parameter adjustments (*α* = 0.5, *β* = 0.5) were implemented to satisfy Scenario 2’s stability requirements. These parameters account for opportunity costs associated with rejecting sustainable production and variations in consumers’ perceived utility of quality products, reflecting enhanced benefits for active innovation and strengthened consumer upgrade willingness. These adjustments fulfil the conditions of inequality system (15), with results presented in Fig 4. Fig 4(a) shows governmental adoption of incentive policies across all initial conditions; Fig 4(b) demonstrates universities’ consistent choice of active sustainable production; Fig 4(c) reveals consumers’ persistent selection of consumption upgrades; while Fig 4(d) presents a three-dimensional visualisation of all parties’ evolutionary strategies, confirming the system’s stabilisation at point (1,1,1).

## 4. Results

**Fig 5:**
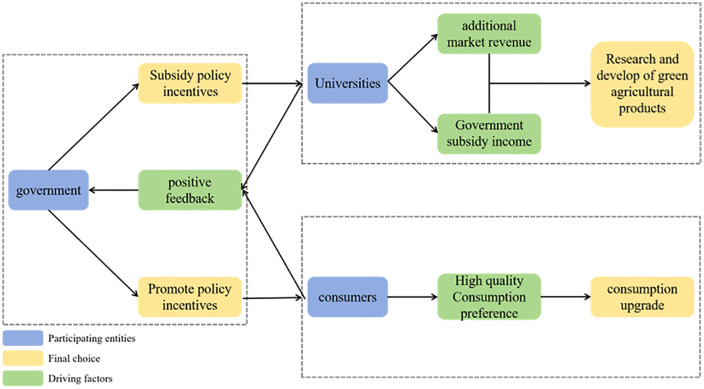
Logical framework for universities’ R&D of green agricultural products.

Scenario analysis confirmed the model’s two evolutionarily stable states at points (1,1,0) and (1,1,1). Initial parameter settings place the system in state (1,1,0), corresponding to digital agriculture’s early development phase in China. This study establishes state (1,1,1) as the target outcome, characterised by government incentives, universities’ active engagement in sustainable agricultural innovation, and consumer adoption of upgraded consumption patterns. A logical framework for universities’ sustainable agricultural production was subsequently developed (Fig 5). Initially, the government employs incentive policies to stimulate both consumption upgrades and universities’ active sustainable production. Upgraded consumption generates market revenue for universities through premium product purchases, creating a collaborative dynamic with the government to encourage sustainable innovation. However, realistic constraints exist: governmental fiscal limitations and consumers’ economic rationality in decision-making. Accordingly, real-world data informed sensitivity analyses of the production subsidy coefficient and consumer preferences for quality-based consumption. The research identifies conditions required for active governmental and consumer participation to achieve Pareto optimal outcomes.

### 4.1. Impact of government subsidy policies on the tripartite

**Fig 6:**
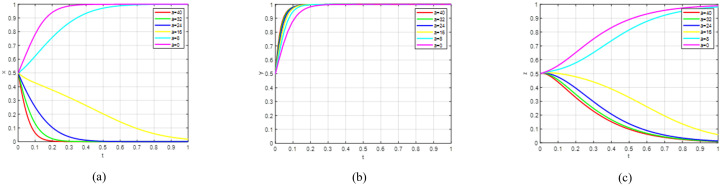
Sensitivity analysis of government subsidy policies on the tripartite. **(a)** Governmental strategic evolution; (b) universities’ behavioural pathway; (c) consumer choice evolution.

Government subsidy strategies to promote the R&D of green agricultural products by universities. To select the subsidy policy, this study sets the university R&D cost subsidy coefficient α as the core explanatory variable, with its value range limited to [0, 1] and discretised in steps of 0.1 to refine the exploration of how variable changes affect the system. Here, α = 0 corresponds to a market state where the government does not intervene in subsidies, while α = 1 represents an extreme intervention scenario where the government fully bears the research costs of universities. Based on this, the government subsidy cost is defined as a = α * Cg₁, where Cg₁ is a fixed parameter with a value of 400. If all other variables remain unchanged, the simulation results are shown in Fig 6.

The results from Fig 6(a)–6(c) indicate that the government subsidy cost (a) exerts a significantly differentiated impact on the strategic choices of the three decision-making entities. When the subsidy cost (a) exceeds the threshold (Cg₃-b), where b = β*Cg₂, the government’s willingness to implement the subsidy policy gradually declines and tends towards 0. As (a) falls progressively below this threshold, the probability of the government maintaining the subsidy policy stabilises, approaching 1. This result demonstrates that rationally controlling the subsidy cost is crucial for ensuring the sustainability of government policy.

The university’s decision to participate in green agricultural product R&D shows a significant positive correlation with the subsidy cost (a). As indicated in the Matlab figure, as the value of (a) increases, the probability of the university choosing to participate in green agricultural product R&D accelerates towards 1. This is because the increase in government subsidy cost reduces the R&D investment pressure on the university, expands its profit margin, thereby creating a strong incentive effect for research.

Consumer purchasing decisions exhibit significant dynamic evolutionary characteristics. When the government subsidy cost is below a critical threshold, purchase intention shows a steady upward climb and gradually approaches 1. Conversely, when the subsidy cost exceeds this threshold, purchase intention displays a continuous downward trend and ultimately tends towards 0. This is because government subsidies play a regulatory role by directly reducing the market pricing scope for green agricultural products. Specifically, when the subsidy intensity reaches a level where the price of green agricultural products becomes comparable to that of traditional ones, while their quality advantage is maintained, consumers—based on the rational decision-making principle of utility maximisation—will gradually establish and reinforce a stable preference for green consumption. Conversely, if insufficient government willingness to subsidise results in a low subsidy amount, while the university’s cost input in the green agricultural product R&D stage remains rigid, the university’s per-unit input cost will be directly increased. To safeguard its own profit level, the university will transfer the cost pressure by raising the selling price of green agricultural products. Simultaneously, if the government fails to effectively guide consumers towards consumption-upgrading awareness through publicity, and consumers have not yet established sufficient recognition of the value of green agricultural products, their decision-making—faced with a significant price differential between traditional and premium-priced green products—will tend to favour low-cost traditional alternatives. This, in turn, leads to persistently low purchase intention for green agricultural products.

### 4.2. Impact of government publicity policies on the tripartite

**Fig 7:**
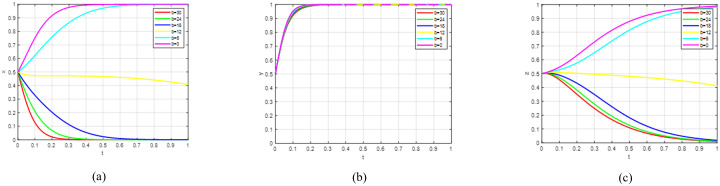
Sensitivity analysis of government publicity policies on the tripartite. **(a)** Governmental strategic evolution; (b) universities’ behavioural pathway; (c) consumer choice evolution.

The government implements publicity strategies to promote consumer upgrading. This study sets the publicity coefficient β as the core explanatory variable, with a value range of [0, 1], and discretises it at intervals of 0.1. When β = 0, it corresponds to the absence of government publicity intervention; when β = 1, it indicates that the government implements a comprehensive, high-intensity publicity investment strategy. Within this framework, the government’s publicity cost is b (b = β*Cg₂). Therefore, with all other variables held constant, the simulation results are shown in Fig 7.

Based on the results in Fig 7(a)–(c), the government’s publicity intensity exhibits significant heterogeneous effects on the strategic choices of the tripartite decision-making entities. When the publicity cost (b) exceeds the threshold (Cg₃ - a), the government’s willingness to implement the publicity policy gradually diminishes and tends towards 0. Conversely, when (b) falls progressively below this threshold, the willingness to implement the policy steadily rises and approaches 1. This result verifies the critical role of appropriately controlling publicity costs in maintaining the stability of government policy supply.

A negative correlation exists between the university’s decision-making behaviour regarding the R&D and production of green agricultural products and the publicity cost (b). That is, as the value of (b) decreases, the probability of the university expanding its production scale accelerates towards 1, although the change is not pronounced. This is because decisions on the production scale of green agricultural products at universities are predominantly governed by rigid factors such as R&D conversion efficiency, production capacity constraints, and teaching and research objectives. Their production relies on specific technologies and equipment, capacity expansion is subject to cyclical limitations, and their decision-making is not solely driven by market profitability but rather balances multiple non-market objectives. Consequently, universities exhibit low sensitivity to market-oriented factors such as publicity cost. Publicity costs can only marginally and slightly influence production decisions, making it difficult to drive significant changes in production scale.

The dynamic evolution of consumer upgraded consumption decisions presents relatively complex characteristics. The simulation results show that the publicity cost (b), as a core input variable for information transmission, exerts a non-linear influence on consumption willingness. At low levels of (b), information asymmetry caused by insufficient information supply suppresses the willingness for consumption upgrading. At high levels of (b), improved information adequacy and enhanced value perception form a synergistic effect, thereby stimulating consumption willingness. This evolutionary pattern essentially reflects the bounded rationality trait in consumers’ green consumption decisions. That is, their decision-making behaviour is not only constrained by intrinsic preferences but also significantly reliant on the external information environment. This ultimately leads to the complex dynamic characteristic of consumption willingness, which exhibits an initial decline followed by an increase.

### 4.3. Impact of green agricultural product prices on the tripartite

**Fig 8:**
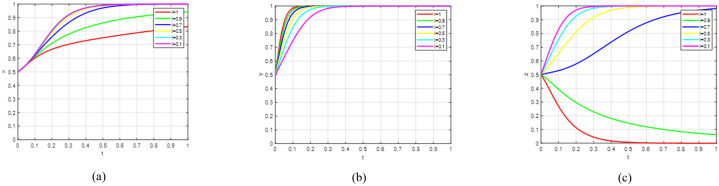
Sensitivity analysis of green agricultural product prices on the tripartite. **(a)** Governmental strategic evolution; (b) universities’ behavioural pathway; (c) consumer choice evolution.

Parameter i, representing consumer preference for premium agricultural produce, ranges from 0 to 1. This variable was discretised using 0.2 increments to accurately assess how preference levels affect market dynamics. A value of i = 0 signifies traditional consumption patterns, whereas i = 1 represents complete preference for consumption upgrading. Analysis revealed a positive correlation between green agricultural product prices (Rm₁) and preference level i. Using a fixed value of P₁*Q₁ = 29, we developed an analytical model to examine price dynamics relative to preference changes. Simulation results under constant conditions are presented in Fig 8. Fig 8(a)–8(c) demonstrate that premium consumption preferences differentially influence the three stakeholder groups’ strategic decisions. A negative correlation exists between preference level and government willingness. That is, when the preference level declines, the government’s willingness to implement policies rises significantly. The core logic of this phenomenon lies in the following chain: the weakening of consumer preference for green agricultural products leads to a failure of the market’s self-regulating mechanism and exacerbates information asymmetry, which in turn undermines the momentum for green agricultural development. To fulfil its public governance objectives of correcting market failures and ensuring the sustainable development of the green agricultural industry, the government needs to strengthen policy intervention to compensate for market deficiencies. Consequently, a decline in preference level propels an increase in government support willingness, which tends towards 1 in the long run to maintain industrial development stability.

A positive correlation exists between preference level and university willingness. Specifically, when the preference level decreases, the university’s willingness regarding both production and R&D declines correspondingly. The underlying mechanism is that consumer preference directly determines the market demand scale and profit expectations for green agricultural products. This serves as a core market signal for universities when conducting technology transfer and making production decisions. However, a weakening preference level induces a contraction in market demand, leading to increased uncertainty regarding the economic return on the university’s R&D investment. This, in turn, reduces its willingness to expand production scale. However, in the long term, owing to its technological R&D advantages and government policy support, the university’s participation willingness will still gradually tend towards 1.

The influence of preference level on consumers’ own willingness for upgraded consumption exhibits a dynamic characteristic of “initial decline, followed by a gradual rise, and finally tending towards 1”. This is because, in the initial stage, a decrease in consumer preference directly weakens the market demand signal for green agricultural products. This leads consumers to reduce their willingness for upgraded consumption due to insufficient value perception. However, with the implementation of external interventions such as government policy guidance and university technology promotion, consumers’ understanding of the ecological and health attributes of green agricultural products gradually deepens. Additionally, social value recognition of green consumption continuously increases. Consequently, consumers’ willingness for upgrading slowly recovers and ultimately tends towards 1, fostering a stable green consumption market environment.

### 4.4. Impact of green R&D costs on the tripartite

**Fig 9:**
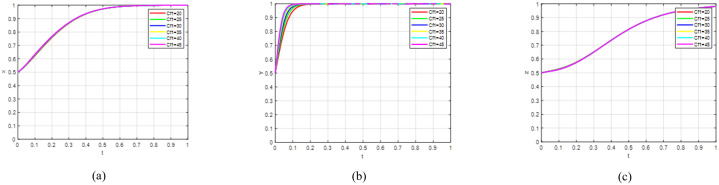
Sensitivity analysis of green R&D costs on the tripartite. **(a)** Governmental strategic evolution; (b) universities’ behavioural pathway; (c) consumer choice evolution.

Green R&D production costs (*Cf₁*) are a critical input for agricultural green transformation, directly influencing the progress and effectiveness of the transition from traditional high-input modes to eco-friendly ones. This study uses a step size of 0.1 for discretisation to precisely capture the impact of changes in green R&D production costs on the market system. With all other variables held constant, the simulation results are shown in Fig 9. The results from Fig 9(a)–(c) indicate that green R&D and production costs exert an influence on the strategic choices of the three decision-making entities. The study finds that the impact of green R&D and production costs on government and consumer decision-making is not pronounced. However, its impact on the university’s production decisions is relatively evident, though the overall trend still converges towards 1.

Regarding the government, whose objective is to maximise social welfare, the positive externality of green R&D leads to insufficient spontaneous market supply. When Cf₁ rises, the degree of market failure intensifies, prompting the government’s willingness to correct this externality through incentive policies to increase rapidly. However, when costs become excessively high, the marginal effect of the policy diminishes, causing willingness to stabilise. This aligns with the logic of public policy for externality correction.

For the university, its decisions follow a “cost–benefit” trade-off process. An increase in Cf₁ directly raises the marginal cost of R&D. When the marginal cost exceeds the marginal revenue, the net return from R&D diminishes rapidly, consequently leading to a decline in R&D willingness. This conforms to the threshold effect of innovation incentives and the input constraint principle for rational agents.

For consumers, their willingness for upgraded consumption is subject to the combined effects of price elasticity, environmental preference, and policy incentives. Specifically, a rise in Cf₁ pushes up the price of green agricultural products through cost transfer, and the substitution effect suppresses consumption willingness. However, the utility gain from environmental preference and the actual cost offset from government consumption subsidies result in a slow rise in consumption willingness. This reflects the synergistic interaction between the income effect and substitution effect for environmentally friendly normal goods.

## 5. Discussion

This research focuses on three core stakeholders: government entities, universities, and consumers. These three parties represent essential components in analysing sustainable agricultural production systems. As policymakers and social resource allocators, governments provide crucial institutional guidance for sustainable agricultural development [32]. Government-formulated incentive policies significantly influence both universities’ technological innovation behaviours and consumer market choices. Universities serve as innovation frontiers [33], integrating research teams and specialised expertise into agricultural production, thereby driving green agricultural transformation. However, they face practical challenges including R&D funding constraints, technology transfer barriers, and limited farmer adoption willingness. As primary demand drivers, consumers shape market structures through preferences and purchasing decisions, significantly constraining universities’ technological and operational decisions. An evolutionary game model was developed to examine how universities can overcome current challenges in leading green agriculture development. Findings reveal that governmental incentives significantly affect both universities’ sustainable development decisions and consumer market behaviour. Secondly, consumer market demand directly influences universities’ technology development and production choices. Finally, initial policy implementation requires critical government subsidies, while long-term development relies more dominantly on market mechanisms’ endogenous incentives. These conclusions suggest that establishing coordinated institutional frameworks combining policy guidance with market regulation [34] is crucial for achieving sustainable agricultural development in China.

This study reveals that the impact of green agriculture on sustainable development exhibits multidimensional characteristics, primarily encompassing the following three dimensions. First, in terms of establishing a green agricultural production technology system, universities leverage the professional advantages of university research teams to integrate green production technologies such as precision fertilisation and biological pest control, thereby establishing a standardised management system covering the entire production chain from planting to harvesting [22]. This approach substantially decreases chemical fertiliser and pesticide application rates, effectively mitigating agricultural non-point source pollution. While safeguarding regional ecosystem integrity, it improves the safety and quality of farm produce, optimises agricultural resource recycling efficiency, and fosters synergistic development between ecological and economic benefits [35]. Taking the Zhejiang Cangnan Blue Crab Science and Technology Backyard as an example, its innovative “mangrove planting–ecological aquaculture coupling” model not only significantly improves blue crab quality but also enhances water quality in surrounding waters through ecological restoration functions and increases benthic biodiversity, providing a typical example for green agricultural production. Second, in terms of theoretical innovation and practical application, this study provides theoretical support and methodological guidance for universities to engage in green agricultural production practices, holding significant value for promoting the green transformation of the agricultural industry and the sustainable development of rural economies. Through the establishment of a routine agricultural science interaction mechanism, universities disseminate green agricultural concepts and cultivate an ecological agricultural development perspective among farmers, while simultaneously conducting technical training and knowledge popularisation. This effectively improves farmers’ production skills, stimulates innovation and development in rural areas, and promotes agricultural industrial upgrading [36]. Simultaneously, the premium value of sustainable agricultural produce, derived from quality superiority, substantially enhances agricultural producers’ economic returns and elevates rural residents’ living standards. This progression effectively reduces regional development disparities between urban and rural areas while facilitating rural revitalisation strategy implementation [37]. Finally, in terms of global sustainable agricultural development practices, universities have established a green agricultural development system with strong adaptability and high operability through technological integration and innovation, institutional model optimisation, and market mechanism cultivation. By engaging in long-term collaborative innovation with farmers, they have developed localised green agricultural technology solutions, which have attracted diverse participation from public and private sectors [38]. This has effectively addressed the technical, financial, and market barriers faced by smallholder farmers in their green transition. This model provides a replicable pathway for sustainable green agricultural development in developing countries, playing a significant role in alleviating global food security pressures, curbing ecological degradation trends, and promoting regional balanced development [39]. Additionally, by leveraging digital technology to upgrade the entire agricultural value chain, universities help enhance the international competitiveness of agricultural products from developing countries, contributing Chinese wisdom to global agricultural emissions reduction, carbon sequestration, and biodiversity conservation, and promoting the construction of a human destiny community where agricultural civilisation and natural ecology coexist in harmony.

## 6. Summary

Green agriculture is the future trend of agricultural development. By leveraging digital technologies such as the Internet of Things, it can enhance the greening and intelligence of agriculture, optimise resource allocation, and strengthen competitiveness and sustainability [40]. As a global agricultural leader, China faces challenges in balancing green agricultural development with socio-economic advancement. However, universities’ green agricultural innovation efforts remain at an early developmental stage, despite multiple stakeholders’ participation. Given universities’ crucial role in achieving quality-led agricultural development, investigating their sustainable agricultural production represents an urgent research priority.

Research on advancing sustainable agricultural innovation and production through universities has been consistently conducted in Western countries.For instance, the study “The Influence of Governmental Agricultural R&D Expenditure on Farmers’ Income-Disparities between EU Member States” establishes through regression modelling that governmental agricultural R&D spending positively influences farmer incomes across EU member states. This finding suggests that universities may utilise R&D funding to optimise technological applications and improve the economic viability of sustainable agricultural production [41]. Meanwhile, China has also introduced relevant policies. For example, in August 2021, the Ministry of Agriculture and Rural Affairs and other departments issued the “14th Five-Year National Plan for Green Development in Agriculture” [42]. The proposal is to create university-affiliated Science and Technology Backyards on the front lines of production, with the objective of providing guidance to scientific research personnel in the dissemination of green technologies. In February 2022, the Ministry of Education, the Ministry of Agriculture and Rural Affairs, and the China Association for Science and Technology jointly issued the “Notice on Promoting the Graduate Student Training Model of Science and Technology Backyard to Support Rural Talent Revitalisation” [43], explicitly proposing to promote the graduate student training model of universities to support rural talent revitalisation.

However, government subsidies function as external incentives with inherent limitations [44], including transient efficacy and limited scope, and cannot reliably stimulate universities’ sustained engagement in green agricultural innovation and production. Consequently, to advance universities’ green agricultural initiatives, governmental support should function as supplementary measures rather than primary drivers.

To stimulate universities’ active engagement in green agricultural innovation and production, greater emphasis must be placed on consumer market mechanisms. Research indicates that evolving consumer demands frequently drive corresponding supply chain transformations. To address consumer preferences for premium agricultural produce, suppliers must pursue technological innovation to deliver quality products to markets [45]. Therefore, if the market is effective, the R&D of green agricultural products by universities will achieve price premiums by improving product quality under the influence of price mechanisms. Nevertheless, China’s present agricultural market remains dominated by conventional consumption patterns. Research by [46] demonstrates that although premium market segments show demand for sustainable agricultural produce, limited consumer awareness results in inadequate supply, thus motivating agricultural enterprises to pursue green product development for potential market needs. This situation persists due to substantial investment requirements for agricultural digital technologies. As rational economic actors, universities primarily assess the advantages and risks associated with developing sustainable agricultural goods through cost-benefit analysis [47]. They are only willing to actively develop and produce green agricultural products when the returns from such activities exceed the costs incurred during the development and production process. Therefore, to leverage incentive mechanisms and achieve consumption upgrading, high-quality consumer demand should be used to drive universities to develop and produce green agricultural products.

Our simulation results demonstrate that both subsidy levels and premium consumption preferences have a significant positive influence on universities’ willingness to engage in green agricultural innovation. Furthermore, market mechanisms function as crucial endogenous incentives driving universities’ sustainable agricultural production.

Collectively, governmental incentive policies and market mechanisms significantly influence universities’ engagement in sustainable agricultural innovation and production. However, their promotional effects vary temporally. Short-term analysis reveals that governmental R&D subsidies critically support universities’ initial sustainable agricultural development. Prospect theory suggests individuals typically exhibit risk aversion, with such aversion positively correlating with decision-makers’ sensitivity to potential losses versus gains [48]. Universities possess distinctive attributes that heighten their sensitivity to potential gains and losses. Consequently, during initial development phases, governmental subsidy policies can mitigate universities’ aversion to preliminary capital investments, thereby enhancing their propensity to actively pursue sustainable agricultural innovation and production [49]. Over the long term, as governmental subsidies phase down, market mechanisms assume greater importance in stimulating universities’ active engagement in sustainable agricultural innovation. Two primary factors explain this transition: First, subsidy programmes may impose significant fiscal pressures. In Hangzhou, for instance, these subsidies cover 50% of equipment procurement costs, substantially adding to governmental fiscal burdens. Second, widespread adoption of digital technologies progressively reduces technical costs associated with sustainable agricultural development. Following initial demonstrations of digital applications, universities recognise substantial performance advantages in active sustainable agricultural innovation. Consequently, even absent governmental support, universities maintain both the capability and motivation to pursue sustainable agricultural production. Furthermore, consumer markets exert a decisive influence on resource allocation processes.Harnessing market mechanisms can effectively stimulate universities’ enthusiasm and intrinsic motivation for sustainable agricultural innovation [50], thereby facilitating their sustainable and healthy development. Consequently, during initial phases of universities’ engagement in sustainable agricultural production, enhanced governmental subsidies and promotional support are essential. For long-term sustainability, strategies should maximise market mechanisms’ role in maintaining universities’ active involvement in green agricultural innovation.
